# Hypoxanthine Induces Signs of Bladder Aging With Voiding Dysfunction and Lower Urinary Tract Remodeling

**DOI:** 10.1093/gerona/glad171

**Published:** 2023-07-18

**Authors:** Lori A Birder, Amanda S Wolf-Johnston, Irina Zabbarova, Youko Ikeda, Anne M Robertson, Ricardo Cardozo, Fatemeh Azari, Anthony J Kanai, George A Kuchel, Edwin K Jackson

**Affiliations:** Department of Medicine, University of Pittsburgh School of Medicine, Pittsburgh, Pennsylvania, USA; Department of Pharmacology and Chemical Biology, University of Pittsburgh School of Medicine, Pittsburgh, Pennsylvania, USA; Department of Medicine, University of Pittsburgh School of Medicine, Pittsburgh, Pennsylvania, USA; Department of Medicine, University of Pittsburgh School of Medicine, Pittsburgh, Pennsylvania, USA; Department of Medicine, University of Pittsburgh School of Medicine, Pittsburgh, Pennsylvania, USA; Department of Mechanical Engineering and Materials Science, University of Pittsburgh, Pittsburgh, Pennsylvania, USA; Department of Mechanical Engineering and Materials Science, University of Pittsburgh, Pittsburgh, Pennsylvania, USA; Department of Mechanical Engineering and Materials Science, University of Pittsburgh, Pittsburgh, Pennsylvania, USA; Department of Medicine, University of Pittsburgh School of Medicine, Pittsburgh, Pennsylvania, USA; Department of Pharmacology and Chemical Biology, University of Pittsburgh School of Medicine, Pittsburgh, Pennsylvania, USA; UConn Center on Aging, University of Connecticut, Farmington, Connecticut, USA; Department of Pharmacology and Chemical Biology, University of Pittsburgh School of Medicine, Pittsburgh, Pennsylvania, USA

**Keywords:** Hypoxanthine, Mitochondrial dysfunction, Oxidative stress, Purines, Urinary incontinence

## Abstract

**Background:**

Lower urinary tract syndrome (LUTS) is a group of urinary tract symptoms and signs that can include urinary incontinence. Advancing age is a major risk factor for LUTS; however, the underlying biochemical mechanisms of age-related LUTS remain unknown. Hypoxanthine (HX) is a purine metabolite associated with generation of tissue-damaging reactive oxygen species (ROS). This study tested the hypothesis that exposure of the adult bladder to HX–ROS over time damages key LUT elements, mimicking qualitatively some of the changes observed with aging.

**Methods:**

Adult 3-month-old female Fischer 344 rats were treated with vehicle or HX (10 mg/kg/day; 3 weeks) administered in drinking water. Targeted purine metabolomics and molecular approaches were used to assess purine metabolites and biomarkers for oxidative stress and cellular damage. Biomechanical approaches assessed LUT structure and measurements of LUT function (using custom-metabolic cages and cystometry) were also employed.

**Results:**

HX exposure increased biomarkers indicative of oxidative stress, pathophysiological ROS production, and depletion of cellular energy with declines in NAD^+^ levels. Moreover, HX treatment caused bladder remodeling and decreased the intercontraction interval and leak point pressure (surrogate measure to assess stress urinary incontinence).

**Conclusions:**

These studies provide evidence that in adult rats chronic exposure to HX causes changes in voiding behavior and in bladder structure resembling alterations observed with aging. These results suggest that increased levels of uro-damaging HX were associated with ROS/oxidative stress-associated cellular damage, which may be central to age-associated development of LUTS, opening up potential opportunities for geroscience-guided interventions.

Aging is associated with pathological changes in the structure and function of both cellular and extracellular components of the lower urinary tract (LUT). In some individuals, these pathological changes converge to produce LUT symptoms and signs including urgency and/or urinary incontinence with impaired bladder contractility with weak stream and increased residual urine, nocturia, and decreased bladder sensation (often resulting in incomplete emptying) (1,2). For example, urinary incontinence is one of the most prevalent health concerns confronting women aged over 60 years of age, affecting up to 55% of older community-dwelling women with 20%–25% experiencing severe symptoms (3,4). These symptoms are often grouped into overlapping clinical syndromes (eg, overactive bladder, underactive bladder, urinary incontinence) with clinical elements collectively referred to as lower urinary tract syndrome or “LUTS.” These storage and voiding symptoms increase in prevalence above the age of 65 years, impairing quality of life for millions of people and creating a substantial burden on health care resources. Despite the prevalence and consequences of these conditions in humans, LUTS continues to be undertreated as there are very few effective therapeutic options. Current therapies have serious side effects that lead to less than 20% of patients remaining compliant with treatments after 6 months (5,6).

The entire continence system is a highly complex network of individual components (eg, pelvic floor muscles, neural system, extracellular matrix, mucosa), each contributing to the goals of maintaining continence during storage, but complete emptying when voiding (3,7). The causes of LUTS are widely recognized to be multifactorial involving complex pathophysiologic processes and numerous risk factors for developing LUTS have been identified. The specific factors that are associated with aging and lead to adult LUTS are not well understood and vary among patients, although likely important contributing factors are: ultrastructural changes in both bladder and urethral muscle with accompanying loss in muscle strength, alterations in sensory processing, increased extracellular matrix, and damaged mucosal integrity (7). Notably, chronic ischemia and associated oxidative stress increases with age, and oxidative stress can have deleterious effects on bladder mucosa, innervation, contraction, and elasticity (8). It has been proposed that oxidative stress in the LUT contributes to age-related LUTS. Nonetheless, our understanding of what causes oxidative stress in the LUT and how this affects individual components of the LUT remains incomplete.

Increased oxidative damage by reactive oxygen species (ROS) can be deleterious to cells and plays a key role in disease progression (9–11). Elevated levels of the purine metabolite hypoxanthine (HX) may exhibit harmful effects in a number of organ systems due to the production of ROS when HX is further metabolized by xanthine oxidase (XO) to xanthine and then to uric acid (12,13). Indeed, both HX and xanthine have been used as biomarkers for tissue hypoxia and oxidative stress, which correlate with disease severity in a number of conditions (14,15). We hypothesize that HX (and xanthine) damage mitochondria via increased ROS production, thus leading to even more ROS production by dysfunctional mitochondria, thus triggering a cascade of oxidative stress. This hypothesis forms the basis of the present study, which examines whether and how exposure of the LUT in adult rodents to uro-damaging HX increases oxidative stress and damages all components of the LUT system in a manner that qualitatively resembles changes seen with aging. Our findings raise the possibility of future geroscience-guided therapies targeting aging-related LUTS.

## Method

### Animals

The Institutional Animal Care and Use Committee of the University of Pittsburgh approved all procedures. The investigation conforms to the *Guide for the Care and Use of Laboratory Animals* published by the U.S. National Institutes of Health (NIH Publication No. 85-23, revised 1996). This study employed female adult sexually mature (3 months old) Fischer 344 (F344) rats (Charles River; Wilmington, MA) treated with vehicle (control group) or HX (10 mg/kg/day for 3 weeks) administered in drinking water. We have tracked the amount of water consumption per day and found no variability (demonstrating consistency) in terms of dosing. We established that this dosing paradigm yields large increases in urinary excretion of HX, which mimic urinary HX levels in LUTS patients (16), thus exposing the LUT to pathologically relevant uro-damaging HX (12,13). A limited number of aged (>25 months old) F344 rats were included. Also included were a group of adult F344 rats treated with vehicle control or adenine (150 mg/kg/day for 1 week) administered in drinking water. This dosing regimen mimics that of an adenine model of chronic kidney disease (CKD).

### Voiding Analysis

Rats were housed (24 hours after 4-hour acclimatization) (16–19) in metabolic cages on an ultrasensitive balance to record void volume and frequency; measurements were averaged for each rat. The light cycle was from 7:00 am to 7:00 pm, and food and water were provided ad libitum. Voided urine was collected in cups attached to force displacement transducers (Grass Technologies, Warwick, RI) connected to a computer (Windaq data acquisition software; DATAQ Instruments Inc., Akron, OH). Voiding frequency (voids per hour), intervoid interval, and volume per void were analyzed. Filling cystometry (17,19,20) was also performed in urethane-anesthetized rats (1.2 g/kg s.q.) to assess additional variables, which included maximum voiding pressure (MVP), threshold pressure for voiding (*P*_thr_), baseline bladder pressure (BP, minimum pressure after voiding), bladder capacity (BCAP), and the frequency of nonvoiding contractions. Bladder compliance (BComp) was estimated from the slope of intravesical volume–pressure relationships. Leak point pressure (LPP), which is the lowest pressure at which urinary leakage occurs in the absence of a detrusor contraction and which is associated with poor bladder contractile performance, and either low or high outflow resistance were assessed as per previously published methods (21,22). External urethral sphincter (EUS) electromyography (EMG) activity was continuously recorded with data acquisition software (AD Instruments, Colorado Springs, CO) using percutaneous epoxy-coated stainless steel wires, per published methods (23,24).

### Western Immunoblotting

Bladder wall preparations were homogenized using Lysing Matrix D in a FastPrep 24 instrument (MP Biomedicals, Solon, OH) in HBSS (5 mM KCl, 0.3 mM KH_2_PO_4_, 138 mM NaCl, 4 mM NaHO_3_, 0.3 mM Na_2_HCO_3_, 0.3 mM Na_2_HPO_4_, 5.6 mM glucose, and 10 mM Hepes, pH 7.4) containing complete protease inhibitor (1 tablet/10 mL, Roche, Indianapolis, IN) and phosphatase inhibitor (Sigma, St. Louis, MO, 1:100). After centrifugation (16 200*g*; 15 minutes at 4°C), the membrane protein fraction was prepared by suspending the membrane pellets in lysis buffer (0.3 M NaCl, 50 mM Tris–HCl [pH 7.6] and 0.5% Triton X-100) and the same concentration of protease inhibitors as above. The suspensions were placed on ice and centrifuged (16 200*g*; 15 minutes at 4°C). The protein concentrations were determined using the Pierce BCA protein assay (Thermo Scientific, Rockford, IL). After denaturation (100°C, 5 minutes) in the presence of Laemmli sample buffer, lysate from each sample was separated on a 4%–15% TGX Stain-Free sodium dodecyl sulfate-polyacrylamide gel electrophoresis (SDS–PAGE) (Bio-Rad Laboratories, Hercules, CA). As a reliable loading control, total protein measurement per sample was determined using Bio-Rad Stain-Free SDS–PAGE gel technology. UV-activated protein fluorescence was imaged on a ChemiDoc MP (Bio-Rad). Proteins were transferred to polyvinylidene fluoride membranes, incubated in 5% (w/v) dried milk dissolved in TBS-T (20 mM Trizma, 137 mM NaCl, 0.1% Tween-20, pH 7.6), rinsed with TBS-T, and incubated overnight at 4°C with primary antibodies. Primary antibody for ectonucleotidase CD39 (rabbit anti-CD39/ENTPD1 14211-1-AP; Proteintech Group, Rosemont, IL) was diluted in TBS-T containing 5% (w/v) milk and the membranes were incubated with a secondary antibody which was donkey anti-rabbit horseradish peroxidase (Advansta, San Jose, CA). Next, membranes were washed and incubated in WesternBright Quantum (Advansta, Menlo Park, CA) and imaged on a ChemiDoc MP (Bio-Rad).

### Protein Carbonylation

Assessment of protein carbonylation was performed with an antibody that detects dinitrophenylhydrazine-derivatized carbonyl groups (OxyBlot Protein Oxidation Detection Kit, S7150; Millipore, Burlington, MA). The volume (intensity) of each protein species was determined and normalized to total protein using Image Lab software (Bio-Rad).

### Caspase Activity

In vivo detection of caspase activity was assessed using FAM-FLIVO (Immunochemistry Technologies, Davis, CA). In brief, rats were injected (jugular) with 100 μL of reagent and after 2 hours rats were sacrificed by cardiac perfusion with phosphate buffered saline (PBS). Bladders were collected, pinned flat but not stretched, fixed for 1 hour in 4% paraformaldehyde, cryoprotected and embedded and frozen in OCT (optimal cutting temperature compound), cryosectioned, and imaged. Cells positive for apoptosis were imaged on an Olympus BX-63 microscope using Olympus CellSens software.

### NAD^+^

Levels of total NAD (coenzyme nicotinamide adenine dinucleotide whereby NAD^+^ is the oxidized form and NADH is the reduced form) and NADH were assayed (Abcam, AB282929). In brief, whole bladder tissues were homogenized in extraction buffer and deproteinized with a 10-kDa spin column. Total NAD was measured in the extract, and NADH was measured in a separate aliquot after decomposition of NAD^+^ at 60°C for 30 minutes. The ratio of NAD^+^/NADH was calculated by subtracting NADH from total NAD levels and dividing by NADH.

### Reactive Oxygen Species

5,5-Dimethyl-1-pyrroline-N-oxide (DMPO; Dojindo Molecular Technologies, Rockville, MD) is a spin-trapping reagent that traps free radicals in protein and the DMPO–protein adduct can be quantified using DMPO-specific antibodies with Western blotting (25). Three injections were given (0.5 g/kg, i.p.; 3, 6, and 18 hours prior to sacrifice). Tissues were homogenized in Chelix-treated PBS (2.68 mM KCl, 1.47 mM KH_2_PO_4_, 137 mM NaCl, and 8 mM Na_2_HPO_4_, pH 7.4) containing 100 μM diethylenetriaminepentaacetic acid. Lysates prepared in XT Sample buffer (Bio-Rad 1610791) were separated by Western immunoblotting (using XT-MOPS running buffer, Bio-Rad 1610788). Trapped free radicals were detected with mouse anti-DMPO antibody (ALX-803-340-C100, 1:1 000, 5% bovine serum albumin; Enzo Life Sciences, Farmingdale, NY). Lipid peroxidation was assessed by enzyme-linked immunosorbent assay for 4-hydroxynonenal (4-HNE, LS-F0039; LSBio, Seattle, WA).

### SLC29 Sodium-Independent Equilibrative Nucleoside Transporter

Bladder mucosa was homogenized in Trizol (Invitrogen, Waltham, MA). Following a chloroform extraction, the RNA was isolated and purified with RNAeasy kit (Qiagen, Germantown, MD) following manufacturer instructions. Bulk RNAseq on total RNA extracted from bladder mucosa was obtained from adult and aged rats (*n* = 4 each, Novogene, Durham, NC). Quality filtered and trimmed raw fastq files were aligned to the rat genome using HISAT2 (version 2.0.5) and counted by FeatureCounts (v.1.5.0-p3) and differential expression for ENT1/2 performed using DESeq2 (1.20.0). Data are expressed as box and whisker plot for the target genes (expressed as log_10_ fragments per kilobase of exon per million mapped fragments or FPKM + 1).

### Adenine Measurement

Urine samples were diluted 1–30 with water, and ^13^C_5_^15^N_5_-adenine (internal standard) was added to each sample. Methods to assess purines, including adenine, have been previously reported (26). In brief, adenine was separated by reversed-phase ultraperformance liquid chromatography (Waters UPLC BEH C18 column, 1.7 µm beads; 2.1 × 150 mm) and quantified by selected reaction monitoring using a triple quadrupole mass spectrometer (TSQ Quantum-Ultra; Thermo Fisher Scientific, Waltham, MA) with a heated electrospray ionization source.

### Bladder Geometric Properties

#### Micro-CT protocol

High-resolution microcomputed tomography (micro-CT) imaging (Skyscan 1272; Bruker Micro-CT, Kontich, Belgium) of intact, unfixed bladders and associated postprocessing were used to assess possible changes in the shape and wall thickness of the bladder due to treatment. Briefly, after bladder harvest a luer-lock-adapted needle was inserted into the urethra and secured using suture ties and remaining urine removed using a 1-mL syringe. The bladder was then submerged in a solution of Hank’s buffer salt solution (HBSS). To inhibit smooth muscle cell contraction, the HBSS contained ethylenediaminetetraacetic acid (chelator), nifedipine (voltage calcium channel blocker), and thapsigargin (SERCA pump inhibitor to prevent reloading of intracellular calcium stores). Micro-CT imaging was performed with a pixel size of 20 μm and rotation step of 0.25 degrees. This high resolution is essential for local stress calculations and for insight into local wall remodeling (27). Both bulk geometric variables (eg, volume, meridonal length) and wall thickness maps were computed from 3D reconstructions using Simpleware ScanIP (Synopsys, Mountain View, CA) in conjunction with Materialize 3-matic (Materialise, Leuven, Belgium). The bladder was preconditioned with 4 cycles of inflation from 5 to 35 mmHg using a programmable syringe pump (BS-8000 from Braintree Scientific, Braintree, MA). The bladder was then inflated to 7 mmHg and mounted in a custom 15-mL tube for micro-CT scanning. PBS-soaked wipes within the tube were used to maintain high humidity within the tube and thereby avoid dehydration-related motion artifacts during imaging. Samples were scanned (8 images taken and averaged) at 50 kV and 200 μA, a rotation step size of 0.3°, scanned 180° around the vertical axis. After the low-pressure scan, the bladder was hydrated for 15 minutes, inflated to 35 mmHg, and micro-CT imaging was performed following the same protocol. Several hundred slices were obtained for each bladder and reconstructed into 3D bladder models in STL format using a 3D-image processing software (Simpleware ScanIP from Synopsys). Geometric parameters of the 3D model, including lumen and abluminal volumes and wall thickness, were measured (3-matic software, Materialise). Each 3D bladder model was manually bisected along the sagittal plane, so the bladder lumen and wall thickness were visible. Four lines were drawn across the bladder lumen, orthogonal to the longitudinal axis. Wall thickness measurements were made along these lines on both sides of the lumen and compared. Heterogeneity in wall thickness was quantified using a wall uniformity index (WUI) defined as the region of the minimum to maximum wall thickness along each line. The value of WUI = 1 for perfect uniformity (thickness is the same on each side) and tends to zero with increasing nonuniformity. To capture a better understanding of thickness variation in the middle region of the bladder away from trigonal area and unfilled dome region, WUI was calculated for the middle 2 lines. 4-Hydroxy-L-proline (hydroxyproline), a component of collagen, was assessed as an indicator of structural changes. In brief, hydroxyproline was detected in hydrolysates by a colorimetric assay (Abcam AB222941, Waltham, MA).

### Statistics

Data were analyzed in GraphPad Prism 6 using Student’s *t* tests (2-tailed) and 1-way analysis of variance followed by appropriate post hoc tests. *p* < .05 was considered significant. Results are expressed as means ± *SEM*. *p < .05; **p < .01; ***p < .001; ****p < .0001.

## Results

**Figure 1.**
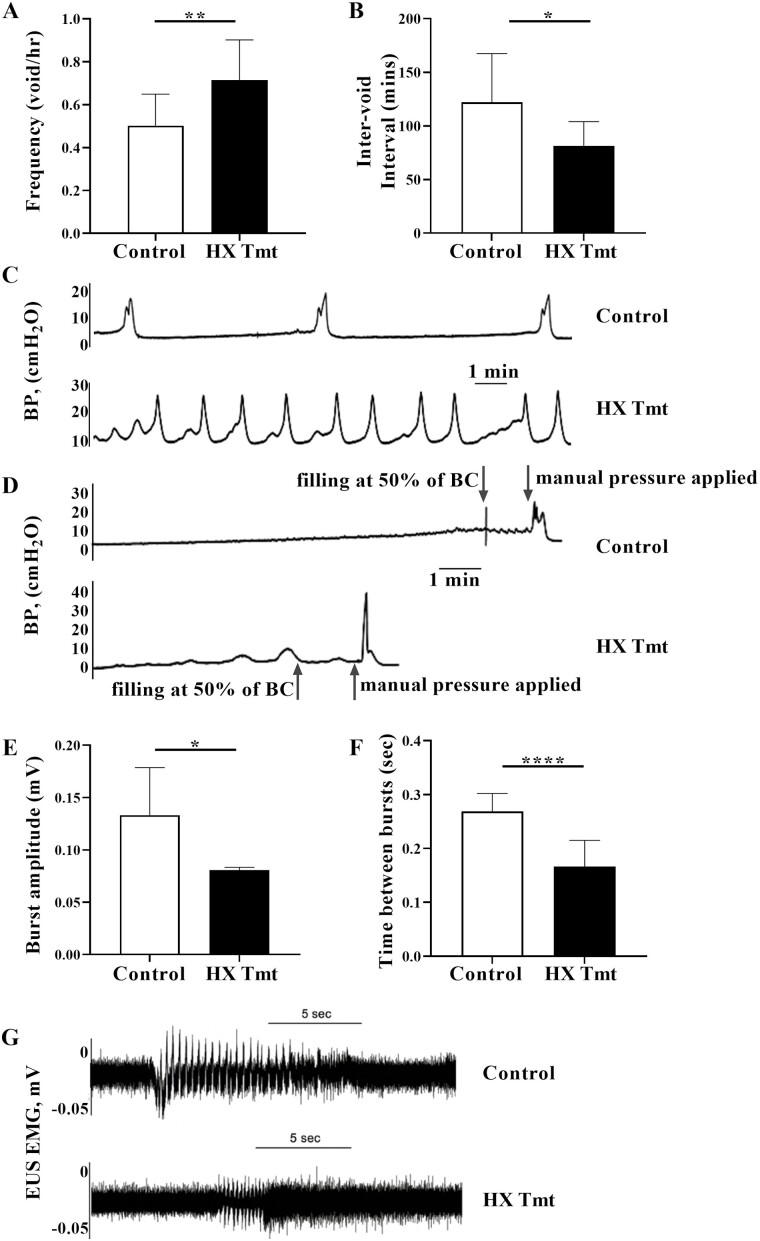
Hypoxanthine (HX) elicited changes in bladder and urethral function. HX (A) increased voiding frequency and (B) decreased the intervoid interval (*n* = 10 each). External urethral sphincter (EUS) electromyography (EMG) activity was recorded and showed that HX decreased both EMG burst (E) amplitude (*n* = 6 each) and (F) frequency (time between bursts; *n* = 10 each). Shown are representative traces depicting (C) bladder voiding function, (D) the leak point pressure at 50% of bladder capacity (BC) (the lowest pressure at which urinary leakage occurs in the absence of a detrusor contraction), and (G) EUS recording from control and HX-treated rats. Data are presented as mean ± *SD*. Ordinary 1-way analysis of variance was used to evaluate significance; *p < .05; **p < .01, ****p < .0001. BP = baseline pressure.

Metabolic-cage studies in HX-treated rats revealed increases in voiding frequency (Figure 1A) and decreases in the intervoid interval (Figure 1B) as compared to untreated, control rats (representative cystometrogram traces shown in Figure 1C). In addition, oral HX treatment decreased the EUS bursting amplitude (Figure 1E) as well as the “silent” period (Figure 1F; representative EMG tracing shown in Figure 1G) (24). These findings indicate inefficient voiding suggestive of urinary incontinence and are also consistent with preclinical and clinical studies showing that bladder irritation results in altered storage and voiding patterns. We also observed that HX-treated rats exhibited significant changes in additional cystometric parameters (Table 1) including: BP, *P*_thr_, MVP, BComp, BCAP, and LPP (at 50% filling, representative trace shown in Figure 1D). In addition, we observed in HX-treated rats spontaneous uninhibited bladder contractions during filling (mean 0.98/min ± 0.13), which were not detected in control, untreated rats. This type of detrusor overactivity is also seen in LUTS patients (28).

**Table 1.** Cystometric Parameters in Control Versus HX-Treated Rats

	BP (cm H_2_O)	*P*_thr_ (cm H_2_O)	MVP (cm H_2_O)	BComp (μL/cm H_2_O)	BCAP (μL)	LPP(50%)
Control	4.1 ± 0.2	6.3 ± 0.5	18.8 ± 1.5	151 ± 19	571 ± 107	28.3 ± 2.4
HX treated	7.8 ± 0.3*	11.1 ± 0.9*	27.5 ± 0.5*	21 ± 7.0*	298 ± 131**	19.6 ± 3.9*

*Notes*: BP = baseline pressure; BCAP = bladder capacity; BComp = bladder compliance; HX = hypoxanthine; LPP = leak point pressure at 50% filling; MVP = maximum voiding pressure; *P*_thr_ = pressure threshold.

*p < .05. **p < .01.

**Figure 2.**
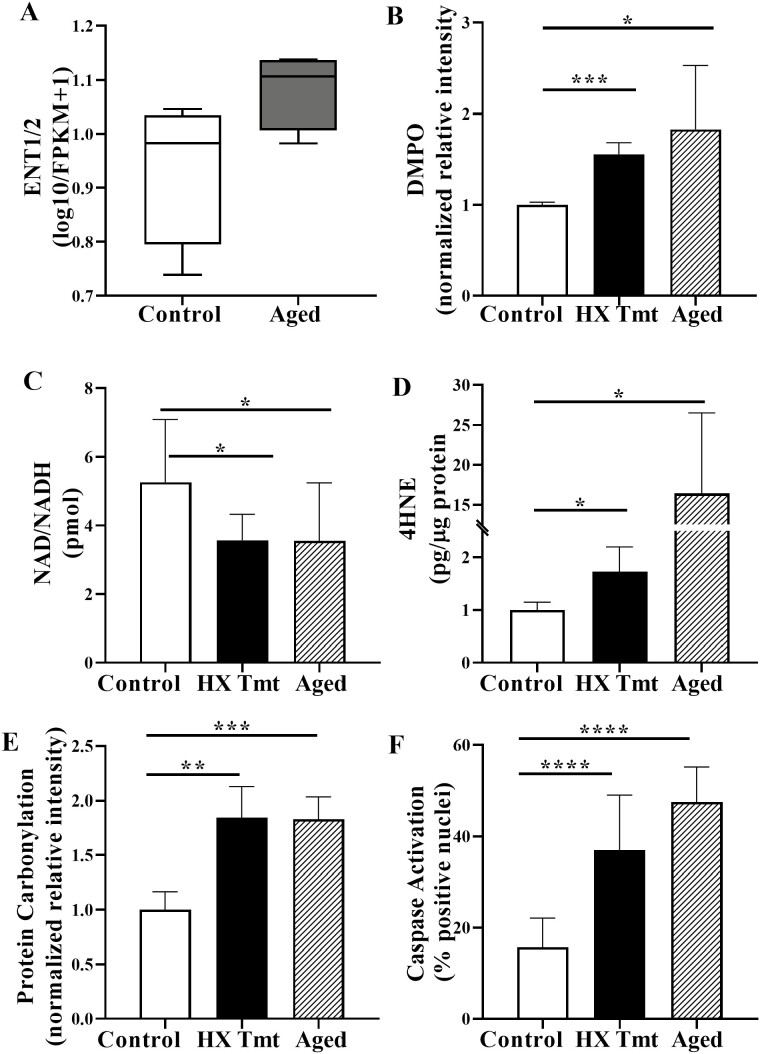
Hypoxanthine (HX) and aging increased oxidative stress and cellular damage. (A) Equilibrative nucleoside transporters (ENT) transport purine and pyrimidine nucleosides. Shown are box and whisker plots depicting increased mean expression of ENT1/2 in aged versus young bladder mucosa; (B) 5,5-dimethyl-1-pyrroline-N-oxide (DMPO) is a spin-trapping reagent that traps free radicals in proteins. Both HX and aging increased protein–DMPO adducts indicating that HX augmented free radical production (*n* = 5 each). (C) nicotinamide adenine dinucleotide (NAD) is a critical coenzyme that influences many key cellular functions and underlies a wide range of aging-related diseases. Both HX and aging decreased the ratio of the oxidized form (NAD^+^) to reduced form (NADH), a decrease in the NAD^+^/NADH ratio causes the enzyme to decrease in activity (*n* = 6 each); (D) both HX and aging increased 4-hydroxynonenal (4-HNE); 4-HNE is a biomarker for lipid peroxidation (*n* = 5 each). (E) Both HX and aging increased protein carbonylation; protein carbonylation is a biomarker for oxidative stress (*n* = 4 each); (F) both HX and aging increased the activation of caspase enzymes; caspases are involved in signaling cascades leading to apoptosis (*n* = 10 each). Data are presented as mean ± *SD*; ordinary 1-way analysis of variance was used to evaluate significance. *p < .05; **p < .01; ***p < .001; ****p < .0001. FPKM = fragments per kilobase of exon per million mapped fragments.

We have previously shown that levels of the free-radical generator HX are significantly elevated in aged rat bladders as compared to young control rat bladders, which is associated with cellular decline (16). We now examined expression of the SLC29 sodium-independent equilibrative nucleoside transporter (ENT) family in rat bladder mucosa. ENT1 and ENT2 participate in the passive transport of purine and pyrimidine nucleosides, and ENT2 also efficiently transports some nucleobases (eg, HX and adenine) along their concentration gradient (12). Aged bladder mucosa exhibited similar increases in ENT1 and 2 (ENT1/2; Figure 2A) versus adult bladder mucosa. A consequence of such increased ENT expression and elevated HX levels in aged bladders would be to increase delivery of HX into cells and exert a negative effect on underlying cells in the bladder wall.

We examined biomarkers of oxidative stress and (patho) physiological ROS pathways. These included measurements of protein adducts with DMPO, a spin-trapping reagent that traps free radicals in protein. DMPO–protein adducts can be localized and quantified using DMPO-specific antibodies with Western blotting. Our findings showed significant increases in endogenous bladder levels of DMPO (Figure 2B) with HX treatment. Even greater increases were also evident in the aged bladder (Figure 2B), showing that this is a valid method to assess oxidative modification in the LUT. Increased oxidative stress can also be detected by depletion of the nucleotide energy metabolite NAD^+^. A decrease in NAD^+^ has been shown to be a function of age (29), consistent with our findings in both the aged and HX-treated bladders (Figure 2C). A consequence of increased oxidative stress is membrane lipid peroxidation. This is consistent with our data that a specific and stable end product of lipid peroxidation product, 4-HNE) (30), was elevated in HX-treated as well as aging rat urinary bladder (Figure 2D). Protein carbonylation (31) is an irreversible state leading to significant damage due to elevated ROS levels. We find that HX increased levels of protein oxidation in HX-treated adult as well as aged bladders (Figure 2E). Our findings demonstrated increased oxidative stress by HX, which, over time, could lead to cell damage and loss of function. Similar to our findings in aged rat bladders, we also observed that HX treatment in adult rats leads to increased caspase activation (Figure 2F), an enzyme involved in signal transduction cascades culminating in apoptosis. Taken together, these new findings add support to link augmented HX with increased oxidative stress and cellular damage.

**Figure 3.**
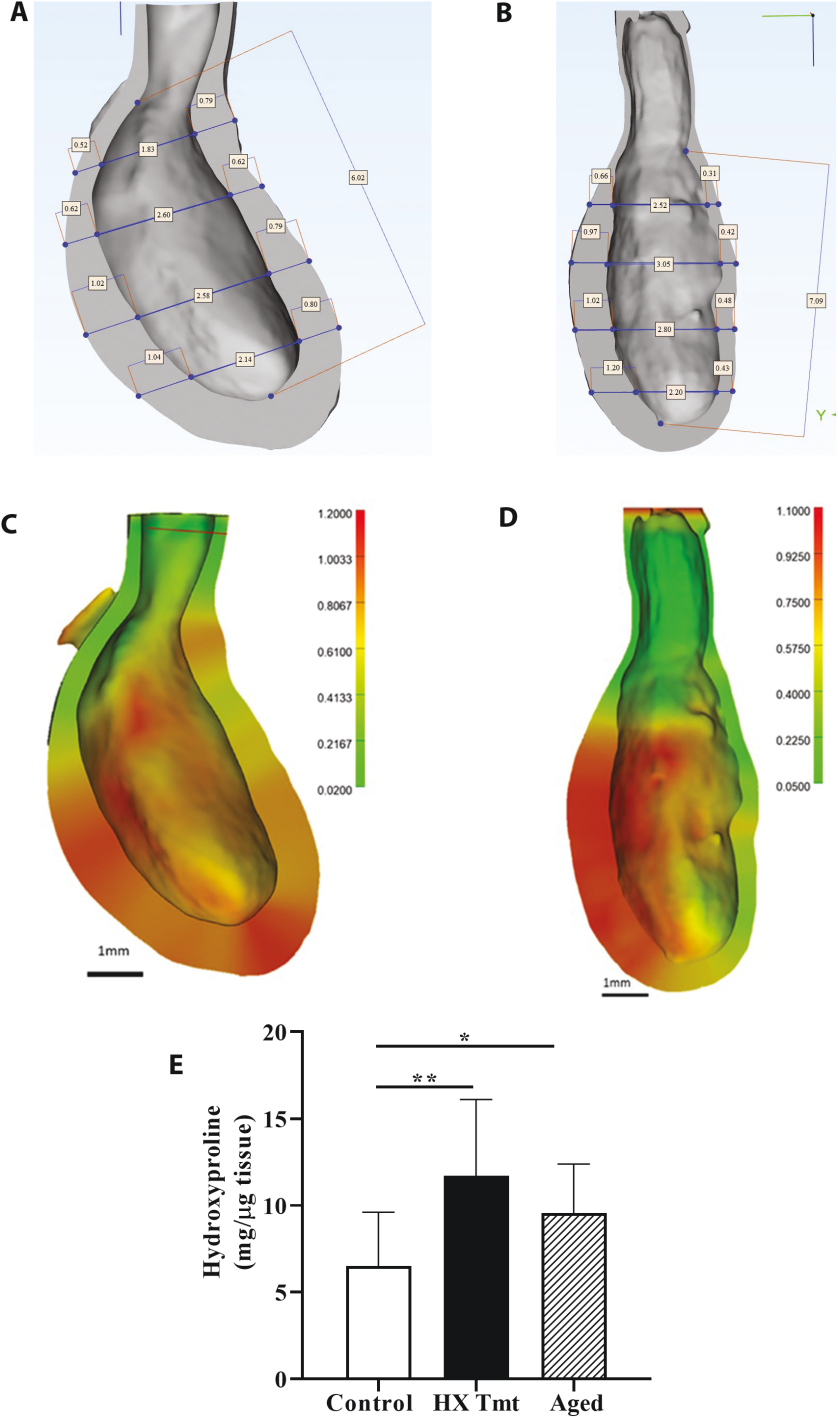
Hypoxanthine (HX) induced bladder wall remodeling. Micro-CT of bisected 3D bladders from (A) control and (B) HX-treated rats was used to evaluate the wall uniformity index as a measure of the uniformity in wall thickness between the anterior and posterior bladder walls. Shown are representative color maps of bladder wall thickness between (C) control and (D) HX-treated rats. Note that HX treatment resulted in asymmetry in bladder wall thickness (*n* = 5 each). (E) HX increased hydroxyproline in the bladder wall, which indicates bladder fibrosis (*n* = 9 each). Data presented as mean ± *SD*; ordinary 1-way analysis of variance was used to evaluate significance. *p < .05; **p < .01. CT = computed tomography.

High-resolution micro-CT is essential for local stress calculations and for insight into local wall remodeling (27). HX treatment induced bladder wall remodeling that led to a statistically significant asymmetry in wall thickness between the anterior and posterior sides of the bladder, visible in the bisected 3D bladder (Figure 3A and B). The WUI is used to quantify the level of uniformity in wall thickness at the same approximate axial position. WUI takes a value of 1 for equal wall thickness and decreases toward 0 as the asymmetry in wall thickness increases. Uniformity in wall thickness was diminished by HX treatment with a decrease in WUI from 0.85 ± 0.11 (untreated) to 0.48 ± 0.18 (treated), *p* < .01 (2 pts per bladder, *n* = 5 for treated and control). This loss of uniformity in thickness is visible in the color maps of wall thickness (Figure 3C and D). Despite this significant difference in WUI, the average wall thickness was nearly identical between control and treated bladders (0.65 ± 0.28 mm vs 0.64 ± 0.20 mm). However, for the HX-treated bladder, the thicker wall is more than 35% greater than this average and the thinner wall is approximately 35% below this average. We suggest that this asymmetry is a maladaptation in response to HX treatment and will contribute to the diminished compliance, which affects voiding efficiency. Along with mechanical and imaging studies, we have evaluated expression of proteins that play a role in ECM organization/remodeling. In this regard, hydroxyproline was significantly elevated in the bladders of HX-treated rats (Figure 3E, 1.6-fold higher in HX-treated rats vs control). Hydroxyproline is associated with collagen and is as an indicator of the severity of fibrosis (32).

**Figure 4.**
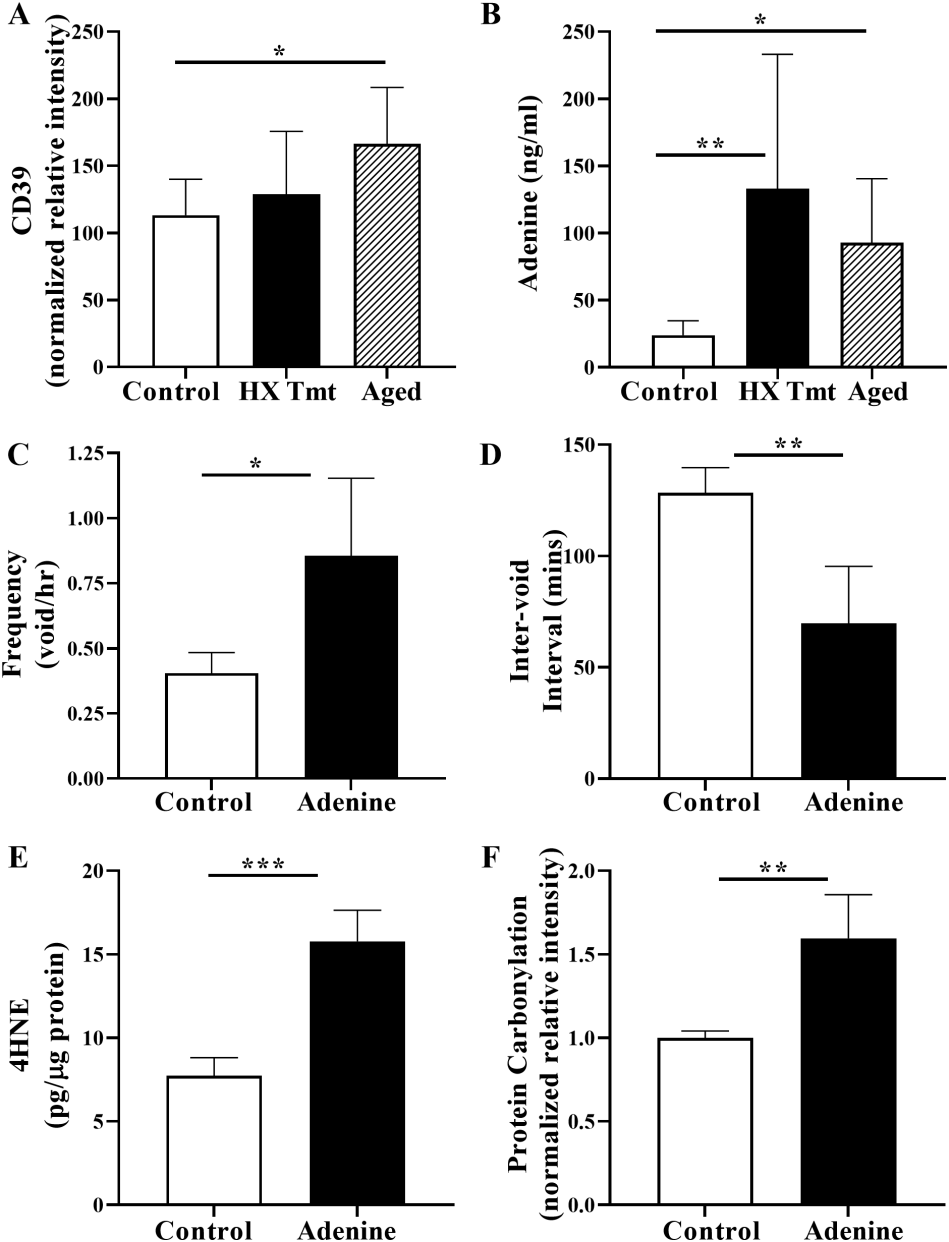
Hypoxanthine (HX) increased urinary adenine and adenine increased bladder injury. (A) Aging increased and HX tended to increase bladder levels of CD39, an enzyme involved in the metabolic pathway leading to adenine (*n* = 6 each). (B) Both HX and aging increased urinary levels of adenine (*n* = 10 each). Oral administration of adenine (C) increases voiding frequency (*n* = 4 each), (D) decreases the intervoid interval (*n* = 4 each), (E) increases bladder levels of 4-HNE, an end product of lipid peroxidation (*n* = 4 each), and (F) increases protein carbonylation, biomarker for oxidative stress in the context of cellular damage (*n* = 4 each). Data presented as mean ± *SD*; ordinary 1-way analysis of variance was used to evaluate significance. *p < .05; **p < .01; ***p < .001. 4-HNE = 4-hydroxynonenal.

The metabolism of adenosine triphosphate (ATP) to adenosine is regulated by the enzyme ENTPD1 (aka CD39), which we find increased with age (Figure 4A). An increase in CD39-mediated ATP metabolism would increase adenosine production, which is also found to increase following inflammation or tissue injury. In turn, adenosine can be converted to its nucleobase adenine via some forms of the enzyme purine nucleoside phosphorylase (PNPase), particularly by bacterial PNPase (33). In both HX-treated and aging rats, urinary levels of adenine were significantly elevated compared to untreated control rats (Figure 4B). Administration of adenine in control rats had no significant effect on plasma creatinine or serum urea nitrate (BUN; not shown), measures used to detect kidney injury. However, we found that adenine administration significantly increased bladder void frequency and decreased the intervoid interval (Figure 4C and Figure 4D, respectively). Adenine-treated rats also exhibit significant increases in both 4-HNE and protein carbonylation (Figure 4E and Figure 4F), measures of protein and lipid oxidation, respectively, that can cause cellular damage and apoptosis and can contribute to injury pathology in the LUT.

## Discussion

Here, we report results comparing urinary bladder form and function in adult rats treated with HX versus untreated controls. Our findings demonstrate that adult rats exposed to HX (mimicking HX levels that we have observed in the urine of LUTS/UI patients, unpublished observations) exhibit significant changes in bladder function and biomarkers for oxidative stress and cellular damage as compared to controls. These findings support our hypothesis that increases in the purine metabolite HX can injure the LUT and thereby render the LUT more susceptible to “triggering” insults, ultimately leading to LUTS. We chose to examine the effects of HX in adult, rather than old, rats because HX is already a likely factor in causing damage in older rats. Therefore, it would be difficult to interpret the damaging effects of HX in already HX-damaged older bladders. Nonetheless, our findings resemble many of the biochemical, structural, and functional changes observed with aging. For example, we have found that both aged- and HX-treated rat bladders exhibit significantly decreased levels of the coenzyme NAD^+^, which is crucial to cellular repair and associated with hallmarks of aging and diseases of aging (34). Thus, these and other observations provide insights into mechanisms whereby biological aging (35) may contribute to age-related dysfunction and diseases of the LUT, offering opportunities for the development of geroscience-guided approaches to LUT aging and diseases (36,37).

Elevated levels of HX may trigger harmful effects due to the production of ROS when HX is further metabolized by XO to xanthine and then to uric acid. HX (via ENTs) (12,13) is efficiently transported across cell membranes, such that increased urinary HX can gain access to underlying cells leading to tissue damage. For example, the HX/XO enzymatic system in the gastrointestinal tract can generate ROS, which in turn can produce gastric lesions and potential hemorrhagic shock. Ischemic conditions are associated with increased production of both HX and xanthine, while antioxidant enzymes are decreased, thus augmenting ROS levels that contribute, for example, to gastric injury (38). The wasting (cachexia) of skeletal muscle in cancer has been linked with HX oxidation to uric acid with increased biomarkers of oxidative stress (39,40). There is also evidence that conditions of muscle atrophy, associated with aging and characterized by loss of muscle mass and contractile capacity, involve increased oxidative stress and mitochondrial dysfunction (40). Although ROS produced by muscle can have physiologic roles, sustained ROS levels result in tissue injury due to oxidative damage and further mitochondrial dysfunction—ultimately resulting in loss of muscle cells, atrophy as well as excessive deposition of collagen fibers and loss of elasticity (40). Indeed, both HX and xanthine have been used as biomarkers for tissue hypoxia and oxidative stress, which correlate with disease severity in a number of conditions (14,15). Not surprisingly, treatments that inhibit oxidation of HX suppress production of inflammatory cytokines and oxidative stress in a number of disorders.

Age-related bladder changes resulting in LUTS are progressive and are associated with both storage and voiding symptoms, indicating multiple defects including alterations in neural function, release of mediators, and defects in smooth muscle and striated muscle structure and function. We used both metabolic cage and urodynamic measurements to assess bladder function (the former could be performed without causing cellular damage due to catheterization). Here, we found that HX treatment significantly increased voiding frequency with a decrease in the intervoid interval. Further, HX treatment resulted in a decrease in the LPP at 50% filling, pressure that is manually applied, results in a leakage of fluid and is used as a surrogate measure to assess urinary incontinence. HX also affected the EUS “silent” period, which is associated with voiding efficiency. We also observed that HX-treated rats exhibited a significant increase in spontaneous uninhibited bladder contractions during filling. This type of detrusor overactivity is also seen in LUTS patients. Our findings are indicative of inefficient voiding often seen with urinary incontinence and are consistent with both preclinical and clinical studies showing that bladder irritation results in altered storage and voiding patterns as reported in LUTS patients (8).

These HX-induced declines in bladder function and outflow tract function could result from a remodeling within component LUT tissues. For example, oxidative stress increases the susceptibility to extracellular matrix remodeling and fibrosis in pelvic floor tissues. Here, we found HX treatment leads to bladder wall remodeling with significant asymmetry in wall thickness and with increased hydroxyproline levels (associated with diseases where fibrosis is a hallmark). Common causes of wall remodeling include inflammation and irritation of the bladder wall, in this case triggered by HX-induced tissue damage. It has been suggested that measurement of detrusor wall shape/thickness could be a useful diagnostic parameter for patients with voiding dysfunction (41). Noninvasive imaging tools that can measure bladder shape changes have been proposed as a clinically useful alternative to conventional urodynamics in terms of identifying involuntary bladder contractions associated with conditions such as overactive bladder or urgency incontinence. To understand the mechanisms by which HX causes alterations in bladder wall geometry, shape, and function, further studies are needed to assess the specific changes to bladder wall components. However, our previous work has revealed that aged rats exhibit significant elevations in (potentially injurious) urinary HX, a free-radical generator, along with decreased blood flow, decreased collagen fiber tortuosity (or fiber waviness), and increased tissue stiffness/reduced BComp (16). Further, treatment of aged rats with a purine analog that inhibits the formation of tissue-damaging HX actually reverses aging-induced tissue damage and dysfunction. Taken together, our findings suggest that elevated urinary levels of HX (possibly as a result of aging or dysregulated purine metabolism) could also contribute to altered bladder mechanical properties resulting in LUTS. Such aging-related synergism via positive feedback loops offers possibilities for the development and validation of future geroscience-guided interventions.

HX-induced injury to the urinary bladder may elevate levels of other purines such as ATP, adenosine, and adenine. ATP plays an essential role in the micturition reflex and is released mostly via the urothelium in response to bladder distension. Elevated levels of ATP have been observed in both LUTS patients and in preclinical models of LUTS/UI as well as in aged rat bladders (42,43). ATP can be rapidly metabolized to adenosine monophosphate (AMP) via CD39, and AMP can be metabolized to adenosine by CD73. CD39 is rate-limiting in this pathway. Importantly, we observed increased expression of CD39 in the urinary bladder of aged rats and rats treated with HX. Adenosine is a signaling nucleoside that is also produced following tissue injury in multiple organs and can elicit both beneficial (anti-inflammatory) and detrimental (fibrosis) effects (44). In turn, adenosine can be metabolized to *adenine* via certain forms of the enzyme purine nucleoside phosphorylase (PNPase), particularly by bacterial PNPases (33). Treatment of rats with HX significantly increases (8.5-fold) urinary *adenine* levels compared to untreated controls. Adenine induces chronic kidney injury in rats and mice and can result in inflammation, fibrosis, and loss of kidney function (45). Further, increased adenine levels also can result in changes in other organ systems, such as the cardiovascular system and the lung (46), where administration of adenine is accompanied by tissue lipid peroxidation, oxidative stress, apoptosis, and fibrosis. We used a modified adenine model used to induce CKD, which, when given for 8 weeks, mimics the slow development of CKD in humans and induces progressive kidney damage with increased plasma creatinine and blood urea nitrogen (BUN) (47). In our studies, treatment of rats with adenine for 1 week did not elicit changes in serum creatinine or BUN yet resulted in significant changes in bladder function with increased voiding frequency and reductions in the intervoid interval and voided volume. Further, we find that adenine results in an increase in both protein carbonylation, as well as 4-HNE, the stable end product of lipid peroxidation due to increased ROS.

## Conclusion

The LUT is very susceptible to the impact of biological aging. Aging-related alterations in LUT structure and function may be caused by multiple factors (eg, peripheral neuropathy and impaired blood perfusion), which could negatively affect the LUT in animals as well as humans. While specific elements of biological aging contributing to LUT dysfunction likely vary among older adults, substantial evidence supports a role for oxidative stress (ie, uncontrolled increases in the production of ROS) in LUTS pathogenesis. Oxidative stress may be produced by conditions such as bladder outlet obstruction, bladder ischemia, and repeated ischemia/reperfusion during a micturition cycle, which can result in complicated changes in both structure and function of the vasculature. In adult rats, the urotoxic purine metabolite HX produces substantial changes in LUT form and function, mimicking many of the changes that are associated with human LUTS. An increase in extracellular HX can result in endothelial dysfunction in part through ROS production and oxidative stress-associated apoptosis. Further, adenine overload can increase pathways of mitochondrial oxidative damage resulting in cell death. Thus, the urotoxic HX, in part by increasing adenine levels, could trigger a cascade of oxidative damage that injures components of the LUT system (including the vascular endothelium), thus further impairing bladder blood flow. Taken together, these and other changes could yield the LUT system prone to dysfunction regardless of the proximal initiating cause and may over time contribute to the development of LUTS in many older adults. Decreasing production of uro-damaging purine metabolites could offer a potentially disease-modifying geroscience-guided approach to preventing the age-associated deteriorations in the LUT, thus potentially enhancing function and independence in older adults by decreasing the burden of LUT diseases.
